# Performance of a Deep Learning Diabetic Retinopathy Algorithm in India

**DOI:** 10.1001/jamanetworkopen.2025.0984

**Published:** 2025-03-19

**Authors:** Arthur Brant, Preeti Singh, Xiang Yin, Lu Yang, Jay Nayar, Divleen Jeji, Yossi Matias, Greg S. Corrado, Dale R. Webster, Sunny Virmani, Anchintha Meenu, Naresh Babu Kannan, Jonathan Krause, Florence Thng, Lily Peng, Yun Liu, Kasumi Widner, Kim Ramasamy

**Affiliations:** 1 Oregon Vision and Pharmacy LLC, Keizer; 2 Google LLC, Mountain View, California; 3 Aravind Eye Hospital, Madurai, India; 4 Verily Life Sciences LLC, South San Francisco, California

## Abstract

**Importance:**

While prospective studies have investigated the accuracy of artificial intelligence (AI) for detection of diabetic retinopathy (DR) and diabetic macular edema (DME), to date, little published data exist on the clinical performance of these algorithms.

**Objective:**

To evaluate the clinical performance of an automated retinal disease assessment (ARDA) algorithm in the postdeployment setting at Aravind Eye Hospital in India.

**Design, Setting, and Participants:**

This cross-sectional analysis involved an approximate 1% sample of fundus photographs from patients screened using ARDA. Images were graded via adjudication by US ophthalmologists for DR and DME, and ARDA’s output was compared against the adjudicated grades at 45 sites in Southern India. Patients were randomly selected between January 1, 2019, and July 31, 2023.

**Main Outcomes and Measures:**

Primary analyses were the sensitivity and specificity of ARDA for severe nonproliferative DR (NPDR) or proliferative DR (PDR). Secondary analyses focused on sensitivity and specificity for sight-threatening DR (STDR) (DME or severe NPDR or PDR).

**Results:**

Among the 4537 patients with 4537 images with adjudicated grades, mean (SD) age was 55.2 (11.9) years and 2272 (50.1%) were male. Among the 3941 patients with gradable photographs, 683 (17.3%) had any DR, 146 (3.7%) had severe NPDR or PDR, 109 (2.8%) had PDR, and 398 (10.1%) had STDR. ARDA’s sensitivity and specificity for severe NPDR or PDR were 97.0% (95% CI, 92.6%-99.2%) and 96.4% (95% CI, 95.7%-97.0%), respectively. Positive predictive value (PPV) was 50.7% and negative predictive value (NPV) was 99.9%. The clinically important miss rate for severe NPDR or PDR was 0% (eg, some patients with severe NPDR or PDR were interpreted as having moderate DR and referred to clinic). ARDA’s sensitivity for STDR was 95.9% (95% CI, 93.0%-97.4%) and specificity was 94.9% (95% CI, 94.1%-95.7%); PPV and NPV were 67.9% and 99.5%, respectively.

**Conclusions and Relevance:**

In this cross-sectional study investigating the clinical performance of ARDA, sensitivity and specificity for severe NPDR and PDR exceeded 96% and caught 100% of patients with severe  NPDR and PDR for ophthalmology referral. This preliminary large-scale postmarketing report of the performance of ARDA after screening 600 000 patients in India underscores the importance of monitoring and publication an algorithm's clinical performance, consistent with recommendations by regulatory bodies.

## Key Points

**Question:**

How does an automated retinal disease assessment (ARDA) algorithm perform clinically in detection of diabetic retinopathy (DR) and diabetic macular edema among more than 600 000 patients in India?

**Findings:**

In this cross-sectional study of 4537 randomly sampled patients with 4537 images, ARDA’s sensitivity and specificity for severe plus DR were 97.0% and 96.4%, respectively. The clinically important miss rate for severe or proliferative DR was 0% owing to misses being graded by ARDA as moderate DR.

**Meaning:**

Results of this study suggest that postdeployment clinical monitoring and publication of all automated algorithms can be recommended to ensure patient safety.

## Teaser

This cross-sectional study evaluates the clinical efficacy of an automated retinal disease assessment algorithm in patients with diabetes.

## Introduction

Diabetic retinopathy (DR) is a leading and growing cause of blindness in India. Screening for DR is important because early identification and treatment can prolong sight. Aravind Eye Hospitals partnered with Google LLC and Verily Life Sciences LLC in 2018 to use a Conformité Européenne (CE)–approved deep learning system for DR screening. This system, an automated retinal disease assessment (ARDA), was used to screen patients with diabetes in India, including those living in remote areas and lacking access to ophthalmologists.^1^ ARDA was developed using more than 130 000 images from the EyePACS dataset and 3 additional datasets from Indian hospitals.^2^ To date, ARDA has screened more than 600 000 patients throughout Tamil Nadu, India.

To assess screening effectiveness, it is important to monitor and report the clinical performance of artificial intelligence (AI) systems,^3^ which can be affected by changes such as acquisition protocol, machine calibration, or drifts in patient population.^4,5^ False-negative findings can result in missed referrals for patients who need specialist care, whereas false-positive findings may result in unnecessary referrals, burdening a resource-constrained health care system. To investigate the postdeployment efficacy of ARDA, we evaluate its clinical performance after screening approximately 600 000 patients.

## Methods

The cross-sectional study was reviewed and approved by the Aravind Eye Hospital Institutional Ethics Committee, and the Declaration of Helsinki^6^ was followed. Written informed consent was obtained by all participants. Reporting adhered to the Strengthening the Reporting of Observational Studies in Epidemiology (STROBE) guideline for cross-sectional studies. This study involved 4537 fundus photographs selected from patients screened between January 1, 2019, and July 31, 2023, across 45 sites. The sites were vision centers, diabetes clinics, or tertiary hospital settings. Patients could present on their own for a comprehensive eye examination or be referred by their primary care physician or endocrinologist. At vision centers, all patients were asked if they had diabetes, and those who had a history of diabetes were recommended to undergo fundus photography. At the vision centers, ophthalmic technicians conducted eye examinations, and cases were reviewed by an ophthalmologist via telemedicine. At the tertiary hospital sites, ophthalmologists always performed comprehensive eye examinations. At the diabetes clinics, comprehensive eye examinations were not conducted; instead, patients underwent fundus photography and ARDA grading. Across all sites, nonmydriatic photographs were initially attempted, with as many as 3 photographs taken per eye. If all photographs were ungradable, patients underwent dilation using 2.5% phenylephrine hydrochloride and 1% tropicamide.

**Figure 1.**
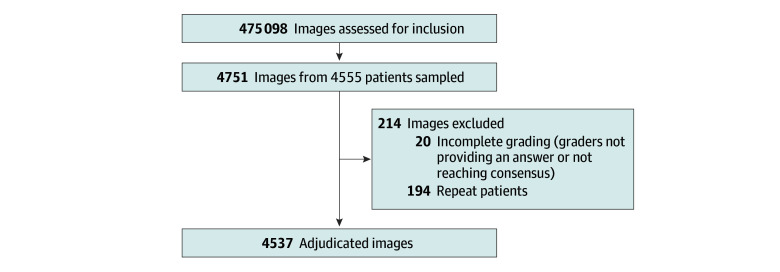
Study Flowchart Patient flowchart includes sampling and filtering for incomplete grading (technical issues) or lack of consensus during adjudication. Approximately 400 images were sampled at random once per calendar quarter across all uses of the algorithm, and these results represent data from the largest partner, Aravind Eye Hospitals.

Selection was performed randomly, approximately once per calendar quarter, and we included images for which all 3 graders reached a consensus (see Figure 1 for details). Images were graded for DR severity in accordance with the International Classification of DR (none, mild, moderate, severe, or proliferative) and the presence of diabetic macular edema (DME), defined as hard exudates within 1 disc diameter of the macula.^7^ Grading and adjudication were performed by 3 graders (from a pool of 20 US board-certified ophthalmologists) per image. Each fundus photograph had metadata for age, sex, laterality, fundus camera (TRC-NW400 [Topcon Healthcare], NFC-700 [CrystalVue], and 3nethra Classic HD [Forus Health]), and clinical site of acquisition. During screening, patients with at least 1 eye indicated by ARDA as ungradable, DR of moderate or greater severity, or DME were referred.

### Statistical Analysis

All statistical analyses were conducted using Python, version 3.11.5 (Python Software Foundation). Summary statistics were used to describe the prevalence of DR and DME as well as demographic characteristics of the study population. For categorical data, χ^2^ tests were performed to determine associations, with a significance threshold of *P* < .05 for 2-sided tests. We estimated 95% CIs for sensitivity and specificity using the Wilson score method. Primary analyses included sensitivity, specificity, positive predictive value (PPV), and negative predictive value (NPV) among gradable photographs for severe nonproliferative DR (NPDR) or proliferative DR (PDR). Secondary analyses measured sight-threatening DR (STDR), defined as severe NPDR, PDR, or DME, and DR and DME gradability. The clinically important miss rate for severe NPDR or PDR and STDR (percentage of patients not referred to an ophthalmologist) was calculated based on referrable DR (defined as moderate NPDR, severe NPDR, PDR, or DME). We further reviewed prior prospective and retrospective studies^8,9,10,11^ and compared the algorithm’s clinical performance with performance in those reports.

## Results

Of the 4537 unique patients with 4537 unique images sampled for adjudication from 45 clinical sites, the mean (SD) age was 55.2 (11.9) years, 2262 (49.9%) were female, and 2272 (50.1%) were male (eTables 2 and 3 in Supplement 1). A total of 1725 patients (38.0%) were from rural sites. Among the DR-gradable photographs (3941 [86.9%]), 3258 (82.7%) had no DR, 126 (3.2%) had mild NPDR, 411 (10.4%) had moderate NPDR, 37 (0.9%) had severe NPDR, and 109 (2.8%) had PDR. A total of 345 of 3715 DME-gradable photographs (9.3%) had DME. This corresponds to 3.7% severe NPDR or PDR and 398 photographs (10.1%) with STDR (Table 1).

**Table 1.** Patient Characteristics

Characteristic	ARDA grades	Grader adjudicated
No. of patients	4537	4537
No. of eyes	4537	4537
Demographic		
Age, mean (SD), y	55.2 (11.9)	55.2 (11.9)
Gender, No. (%)		
Female	2262 (49.9)	2262 (49.9)
Male	2272 (50.1)	2272 (50.1)
Rural, No. (%)	1725 (38.0)	1725 (38.0)
Not gradable, No. (%)		
DR	947 (20.9)	596 (13.1)
DME	983 (21.7)	822 (18.1)
Retinopathy or maculopathy finding, No./No. gradable (%)		
No DR	2652/3590 (73.9)	3258/3941 (82.7)
Mild NPDR	264/3590 (7.4)	126/3941 (3.2)
Moderate NPDR	417/3590 (11.6)	411/3941 (10.4)
Severe NPDR	123/3590 (3.4)	37/3941 (0.9)
PDR	134/3590 (3.7)	109/3941 (2.8)
DME	463/3554 (13.0)	345/3715 (9.3)
Severe NPDR plus PDR	257/3590 (7.2)	146/3941 (3.7)
STDR^a^	489/3590 (13.6)	398/3941 (10.1)

Abbreviations: ARDA, automated retinal disease assessment; DME, diabetic macular edema; DR, diabetic retinopathy; NPDR, nonproliferative DR; PDR, proliferative DR; STDR, sight-threatening DR.

^a^ Defined as severe NPDR, PDR, or DME.

The algorithm’s sensitivity and specificity among gradable photographs for severe NPDR or PDR were 97.0% (95% CI, 92.6%-99.2%) and 96.4% (95% CI, 95.7%-97.0%), respectively. The 4 patients (2.7%) with severe NPDR or PDR that were understaged were graded by ARDA as moderate NPDR. Hence all cases with severe NPDR or PDR were still referred to the clinic, and the clinically important miss rate for severe NPDR or PDR was 0%. PPV and NPV were 50.7% and 99.9%, respectively (as context, prevalence of severe NPDR or PDR was 3.7%) (eTables 1-3 in Supplement 1).

**Figure 2.**
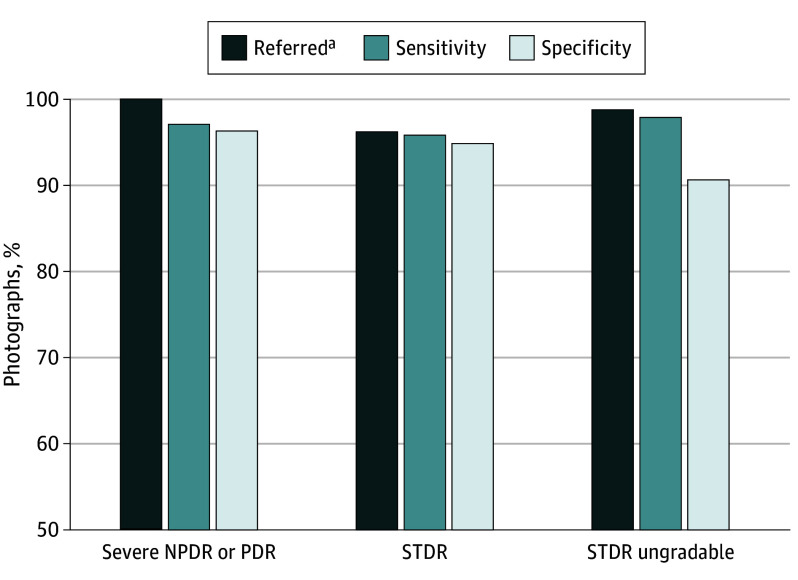
Algorithm Performance Performance is shown for photographs referred, sensitivity, and specificity for severe diabetic retinopathy (DR; severe nonproliferative DR [NPDR] or proliferative DR [PDR]), sight-threatening DR (STDR; defined as severe NPDR, PDR, or diabetic macular edema [DME]), and STDR ungradability. The sample size consisted of 4537 images from 4537 patients. Ground truth labeling categorized 146 photographs as severe NPDR plus PDR, 398 as STDR, and 596 as ungradable STDR. ^a^Referrals can be due to other reasons than the diagnosis category. For example, of severe NPDR or PDR cases, sensitivity was 97.0% (95% CI, 92.6%-99.2%), but all severe NPDR and PDR cases were referred (eg, due to being graded as moderate NPDR, DME, or ungradable), so 100% were referred. Imperfect referral sensitivity of STDR relative to severe NPDR or PDR indicates misses that were adjudicated as DME.

The algorithm’s sensitivity and specificity for STDR were 95.9% (95% CI, 93.0%-97.4%) and 94.9% (95% CI, 94.1%-95.7%), respectively, and the clinically important miss rate for STDR was 3.7% (Figure 2). STDR PPV and NPV were 67.9% and 99.5%, respectively (STDR prevalence was 10.1%). Ungradability was associated with male sex (22.2% male [504 of 2272] vs 19.5% female [442 of 2262]; *P* = .03) and being older than 60 years (32.5% [499 of 1535] for >60 years vs 14.9% [448 of 3002] for ≤60 years; *P* < .001) for both ARDA and adjudication.

## Discussion

ARDA performed well in this cross-sectional study in a clinical setting in India. Using ophthalmologist-adjudicated grades as the reference, ARDA achieved 97.0% sensitivity and 96.4% specificity for severe NPDR or PDR and 95.9% sensitivity and 94.9% specificity for STDR. Performance for STDR and referrable DR was similar to that in prior retrospective and prospective studies using the same deep learning system (Table 2).^8,9,10,11,12^

**Table 2.** Performance Comparison With Prior Prospective and Retrospective Studies

Source	Country	Patient age, mean, y	Female, %	Camera, manufacturer and model	No. of images/patients	Design	Metric	Sensitivity, %	Specificity, %
Gulshan et al,^2^ 2016	US	54	62	CenterVue DRS; iCam Optovue; CR1, DGi, or CR2 Canon; NW Topcon^a^	9963/4997	Retrospective	Referrable DR	90.3 for operating point 1; 97.5 for operating point 2	98.1 for operating point 1; 93.4 for operating point 2
Gulshan et al,^2^ 2016	France	58	43	CenterVue DRS; iCam Optovue; CR1, DGi, or CR2 Canon; NW-400 Topcon^a^	1748/874	Retrospective	Referrable DR	87.0 for operating point 1; 96.1 for operating point 2	98.5 for operating point 1; 93.9 for operating point 2
Ruamviboonsuk et al,^10^ 2019	Thailand	61 (SD, 11)	69	3nethra classic HD Forus Health; CR2 Canon; VX-10, VX-20, Nonmyd 7, Nonmyd WD, Nonmyd α-D III 8300 Kowa; AFC-210, AFC-230, AFC-300 Nidek; TRC-NW-8 Topcon Healthcare; Visucam 200 Zeiss	7517/29 985	Retrospective	(1) Moderate or worse DR; (2) DME	(1) 96.9; (2) 95.2	(1) 95.6; (2) 98.2
Gulshan et al,^12^ 2019	India	57 (SD, 9)	42	3nethra Classic HD Forus Health	997/1983	Prospective	(1) Moderate or worse DR; (2) DME	(1) 88.9 for model 1 and 90.8 for model 2; (2) 97.4 for model 1 and 98.1 for model 2	(1) 92.2 for model 1 and 95.0 for model 2; (2) 90.7 for model 1 and 85.0 for model 2
Gulshan et al,^12^ 2019	India	56 (SD, 10)	33	3nethra Classic HD Forus Health	2052/3779	Prospective	(1) Moderate or worse DR; (2) DME	(1) 92.1 for model 1 and 93.0 for model 2; (2) 93.6 for model 1 and 98.6 for model 2	(1) 95.2 for model 1 and 97.8 for model 2; (2) 92.5 for model 1 and 88.1 for model 2
Limwattanayingyong^9^ 2020	Thailand	First screening: 57 (SD, 11); second screening: 57 (SD, 10)	69 (First screening); 69 (second screening)	TRC-NW8 Topcon Healthcare; AFC-210, AFC-230 Nidek; Nonmyd α-DIII 8300, Nonmyd 7, VX-10α, Nonmyd α-DIII, Nonmyd WX, VX-20 Kowa	5738/NA first screening; 4148/NA second screening	Retrospective, longitudinal (cohort for 2 consecutive screenings over 2 y)	STDR	Screening 1: 95.0; screening 2: 90.1	Screening 1: 98.0; screening 2: 97.9
Ruamviboonsuk^8^ 2022	Thailand	60 (SD, 10)	63	Maestro 3D OCT-1, TRC-NW300 Topcon Healthcare; AFC-230, AFC-210, AFC-300 Nidek	7651/NA	Prospective	STDR	91.4	95.4

Abbreviations: DME, diabetic macular edema; DR, diabetic retinopathy; STDR, sight-threatening diabetic retinopathy.

^a^ Camera models were not further broken down by data source.

Identifying advanced DR is critical because half of untreated eyes with severe PDR develop blindness within 5 years, and retinal photocoagulation and anti–vascular endothelial growth factor therapy significantly decrease the chance of blindness.^13^ ARDA’s clinical miss rate for patients with severe NPDR or PDR was 0% because all these patients were detected as having at least moderate NPDR and referred to clinic. We estimate that at least 22 200 patients with severe NPDR or PDR have been referred since 2019 based on the observed rate and the 0% clinically important miss rate. ARDA’s DR operating point prioritized sensitivity, yielding an NPV of 99.9% and PPV of 50.7%. Given that severe NPDR or PDR is rare (3.7% prevalence), this PPV indicates a 14-fold enrichment and this sensitivity and specificity tradeoff contributed toward the 0% clinically important miss rate. However, understanding each institution’s optimal trade-off based on resourcing and policies is important future work.

Ungradability remains a challenge in clinical practice, imposing significant costs on both patients and health care systems. Older age is the greatest risk factor (3-fold) for ungradability, possibly due to increased prevalence of media opacity and incomplete mydriasis. Because the anterior segment photos were not captured in this clinical study, the exact causes of ungradability could not be quantified. ARDA’s gradability model operating point was set to prioritize the diagnostic accuracy of the downstream DR model by providing higher-quality images, though this can in principle be adjusted per institution, similar to the discussion above.

The use of deep learning in computer-aided diagnosis is a rapidly growing field with the first device (IDx-DR; Digital Diagnostics) being cleared by the US Food and Drug Administration (FDA) in 2018 and Verily Life Sciences LLC announcing its CE Mark for ARDA in 2019. Because camera environments, machine calibration, and patient population drift can influence algorithm performance, clinical monitoring is essential to ensure that computer-aided diagnostics remain safe and efficacious. In the US, 3440 instances of the *Current Procedural Terminology* code for AI-backed screenings (92229) have been recorded for any FDA-approved DR algorithm from 2019 to 2023 in the TriNetX database, which encompasses 107 million patients^14^; clinical performance would help with understanding whether any drifts in algorithmic performance might have occurred. Furthermore, deploying model updates, while intended to improve performance, still may inadvertently degrade performance. To the best of our knowledge, we report the first large-scale postdeployment clinical performance analysis for any CE- or FDA-approved ophthalmology AI device. Although the process is costly, we recommend that AI medical device companies publish clinical performance data. Monitoring algorithmic performance is essential to ensure patient safety.

### Strengths and Limitations

Our study’s strengths include data captured in a clinical setting, including rural regions where the need for ophthalmology services is greatest; no exclusion of other diseases with similar features, such as age-related macular degeneration, hypertensive retinopathy, and vascular occlusions; a large clinical sample size of 4537 eyes; and 3 different fundus camera manufacturers. Limitations include a population enriched for a single ethnicity in Tamil Nadu, which may not be fully representative of the entire Indian population; defining DME based on exudates on fundus photography (known to be suboptimal compared with optical coherence tomography, but which is not available in clinical screening settings); and using cameras with 45° fields, which may understage predominantly peripheral pathology.

## Conclusions

In this cross-sectional study, we found that ARDA performed well in a clinical screening setting. The sensitivity and specificity for severe NPDR and PDR were high, and the clinically important miss rate was 0%, meaning that 100% of patients with severe NPDR or PDR were referred to an ophthalmologist. We believe this is the first large-scale postmarketing performance report for an AI algorithm in ophthalmology. To ensure continued patient safety, we recommend that all AI algorithms monitor and publish their clinical performance.

## Notes

Supplement 1. **eTable 1.** Confusion Matrix **eTable 2.** Male Patient Characteristics **eTable 3.** Female Patient Characteristics

Supplement 2.
Data Sharing Statement
