# Errors in Results

**DOI:** 10.1001/jamanetworkopen.2025.11258

**Published:** 2025-04-14

In the Original Investigation titled “Performance of a Deep Learning Diabetic Retinopathy Algorithm in India,”^1^ published March 19, 2025, select data in the last sentence of the Results were incorrect: 2 percentages were incorrect, 1 denominator was incorrect, and 2 data groupings were inadvertently transposed. This article has been corrected.
