# Supplementary material for: Performance of a Deep Learning Diabetic Retinopathy Algorithm in India
Source: JAMA Netw Open. 2025 Mar 19;8(3):e250984. doi: 10.1001/jamanetworkopen.2025.0984 (PMC11923701; doi:10.1001/jamanetworkopen.2025.0984)
Supplement: Supplement 1. — eTable 1. Confusion Matrix eTable 2. Male Patient Characteristics eTable 3. Female Patient Characteristics [file jamanetwopen-e250984-s001.pdf]

Supplementary Online Content

Brant A, Singh P, Yin X, et al. Performance of a deep learning diabetic retinopathy algorithm in India. *JAMA Netw Open.* 2025;8(3):e250984. doi:10.1001/jamanetworkopen.2025.0984

eTable 1. Confusion Matrix
eTable 2. Male Patient Characteristics
eTable 3. Female Patient Characteristics

This supplementary material has been provided by the authors to give readers additional information about their work.

© 2025 Brant A et al. *JAMA Network Open*.

eTable 1. Confusion Matrix

A. Severe+

	Reference		
Prediction	Severe+	No Severe+	Ungradable
Severe+	132	125	0
No Severe+	4	3317	12
Ungradable	0	0	0

* Severe+ Diabetic Retinopathy (DR) was defined as severe nonproliferative DR or proliferative DR

**Images labeled as DR Ungradable were excluded from the performance matrix, as 100% of ungradable images were referred to clinic.

B. STDR

	Reference		
Prediction	STDR	No STDR	Ungradable
STDR	514	155	5
No STDR	22	2287	7
Ungradable	0	0	0

* Sight Threatening Diabetic Retinopathy (STDR) was defined as severe NPDR, proliferative DR, or DME Prevalence

**Images labeled as STDR Ungradable were excluded from the performance matrix, as 100% of ungradable images were referred to clinic

© 2025 Brant A et al. *JAMA Network Open*.

C. DME

	Reference		
Prediction	DME	No DME	Ungradable
DME	317	126	20
No DME	20	3025	46
Ungradable	0	0	0

**Images labeled as DME Ungradable were excluded from the performance matrix, as 100% of ungradable images were referred to clinic

eTable 2. Male Patient Characteristics

	ARDA Grades	Grader Adjudicated
**Patients (N)**	2272	
**Eyes (N)**	2272	
**Patient Demographics**		
Age, mean (sd), years	56.2 (11.8)	
Female N (%)	0 (0.0%)	
Rural N (%)	764 (33.6%)	
**Gradability**		

DR Ungradability	504 (22.2%)	327 (14.4%)
DME Ungradability	529 (23.3%)	453 (19.9%)
**Retinopathy or Maculopathy**		
No DR N (% of gradable)	1185 (67.0%)	1497 (77.0%)
Mild NPDR N (% of gradable)	156 (8.8%)	82 (4.2%)
Moderate NPDR N (% of gradable)	250 (14.1%)	263 (13.5%)
Severe NPDR N (% of gradable)	85 (4.8%)	27 (1.4%)
Proliferative DR N (% of gradable)	92 (5.7%)	76 (3.9%)
DME N (% of gradable)	289 (16.6%)	220 (12.1%)
Severe+ N (% of gradable)	177 (10.0%)	103 (5.3%)
STDR N (% of gradable)	308 (17.4%)	254 (13.1%)

* Severe+ was defined as severe NPDR or proliferative DR

** STDR was defined as severe NPDR, proliferative DR, or DME Prevalence

eTable 3. Female Patient Characteristics

	ARDA Grades	Grader Adjudicated

© 2025 Brant A et al. *JAMA Network Open*.

**Patients (N)**	2262	
**Eyes (N)**	2262	
**Patient Demographics**		
Age, mean (sd), years	54.1 (11.8)	
Female N (%)	2262 (100%)	
Rural N (%)	961 (42.5%)	
**Gradability**		
DR Ungradability	442 (19.5%)	269 (11.9%)
DME Ungradability	453 (20.0%)	368 (16.3%)
**Retinopathy or Maculopathy**		
No DR N (% of gradable)	1465 (80.5%)	1758 (88.2%)
Mild NPDR N (% of gradable)	108 (5.9%)	44 (2.2%)
Moderate NPDR N (% of gradable)	167 (9.2%)	148 (7.4%)
Severe NPDR N (% of gradable)	38 (2.1%)	10 (0.5%)
Proliferative DR N (% of gradable)	42 (2.3%)	33 (1.7%)
DME N (% of gradable)	174 (9.6%)	125 (6.6%)
Severe+ N (% of gradable)	80 (4.4%)	43 (2.2%)

© 2025 Brant A et al. *JAMA Network Open*.

STDR N (% of gradable)	181 (9.9%)	144 (7.2%)

* Severe+ was defined as severe NPDR or proliferative DR

** STDR was defined as severe NPDR, proliferative DR, or DME Prevalence

© 2025 Brant A et al. *JAMA Network Open*.
