# Supplementary material for: Performance of a Deep Learning Diabetic Retinopathy Algorithm in India
Source: JAMA Netw Open. 2025 Mar 19;8(3):e250984. doi: 10.1001/jamanetworkopen.2025.0984 (PMC11923701; doi:10.1001/jamanetworkopen.2025.0984)
Supplement: Supplement 2. — Data Sharing Statement [file jamanetwopen-e250984-s002.pdf]

Data Sharing Statement

Data
Data available: No

Additional Information
Explanation for why data not available: Confidential
